# Post-Bonding Crack-Induced Di-Cantilever Bending (PBC-DCB): A Novel Method for Quantitative Evaluation of Bonding Strength for Wafer-to-Wafer and Die-to-Wafer Hybrid Bonding

**DOI:** 10.3390/mi16121304

**Published:** 2025-11-21

**Authors:** Tianze Zheng, Yuan Xu, Cong Mei, Yingjie Chen, Liu Chang, Gangli Yang, Qiuhan Hu, Tailong Shi, Yuan Yuan, Zongguang Yu, Liyi Li

**Affiliations:** 1 School of Integrated Circuits, Southeast University, Wuxi 214000, China; 220236463@seu.edu.cn (T.Z.); 220226140@seu.edu.cn (Y.X.); 230229587@seu.edu.cn (C.M.); siriusirs@163.com (Y.C.); 230239483@seu.edu.cn (L.C.); 230238438@seu.edu.cn (G.Y.); 101013381@seu.edu.cn (T.S.); 2 School of Advanced Technology, Xi’an Jiaotong-Liverpool University, Suzhou 215123, China; qiuhan.hu23@student.xjtlu.edu.cn; 3 The 58th Research Institute of China Electronics Technology Group Corporation, Wuxi 214000, China; 15629070655@163.com (Y.Y.); yuzg58@163.com (Z.Y.)

**Keywords:** hybrid bonding, bonding strength, fracture mechanics, di-cantilever bending

## Abstract

To address the challenge of characterizing bonding strength in hybrid bonding, this paper proposes a method based on post-bonding crack-induced di-cantilever bending (PBC-DCB). The commonly used industrial blade insertion (BI) method only measures the edge strength of wafer-to-wafer samples via bevel geometry, failing to characterize the wafer center or die-to-wafer samples (as they lack a bevel) and exhibiting limited accuracy due to irregular crack edges. This study develops a di-cantilever beam test initiated by a specially prepared post-bonding notch. By deriving the relationship between load–displacement and bonding strength based on fracture mechanics and optimizing sample preparation and testing parameters, quantitative measurement of interface fracture strength is achieved. Experimental results show the method has a deviation < 5% from BI with better repeatability, verifying its reliability. It successfully distinguishes bonding strength between wafer center and edge and reveals that SiCN dielectric layers have 17.34% higher strength and better uniformity than SiO_2_. This provides a reliable testing tool for hybrid bonding quality and process optimization, with broad prospects in industrial production and scientific research.

## 1. Introduction

With the increase in AI demands, a high computing and storage power requires the transmission of massive amounts of data between memory dies and the processor at very high throughput. The dynamic storage and data transfer rates are increasingly unable to keep up with the data processing speed of processors. Therefore, the bandwidth of interconnects between computing dies and memory dies has become a bottleneck for computing power evolution. Die-to-die interconnect density—defined as the number of interconnects per mm^2^ or the minimum pitch—is one of the key factors that determines the bandwidth. The major type of die-to-die interconnects is microbump interconnect. It has been widely recognized that microbumps reach their minimum pitch at around 20 μm.

Hybrid bonding (HB) is a promising technology that has been demonstrated to be capable of breaking the 20 μm pitch limit. Hybrid bonding technology is thus considered an indispensable technology for AI applications [1,2,3,4,5,6,7,8,9]. Due to its low thickness, HB also provides low latency and low power consumption interconnects, effectively solving the “data transfer bottleneck” in computing power enhancement [10,11].

Compared to microbump interconnection, achieving high mechanical strength is one of the major manufacturing challenges during the HB process. The strength of the microbump interconnection depends on the soldering of metal and polymer glue from underfill materials. In contrast, that of HB interconnection depends on atomic diffusion and covalent bonding, which is very susceptible to trace amounts of surface contamination and composition variation at an atomic level. The quantitative measurement of the bonding strength is pivotal in HB processes development and manufacturing quality control.

**Figure 1:**
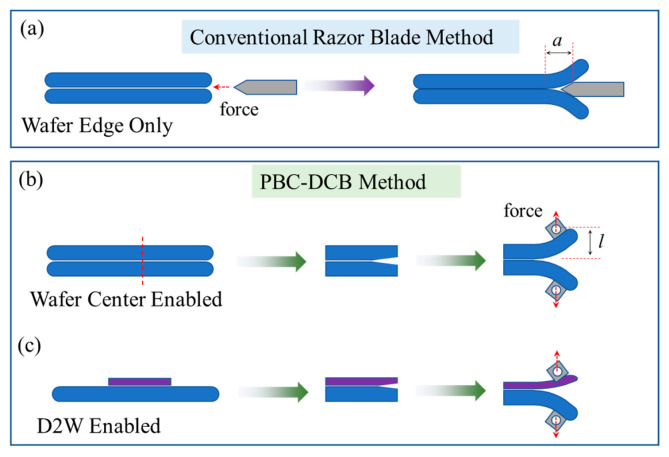
Schematic diagram of the traditional blade method and PBC-DCB process. (**a**) conventional razor blade method. (**b**) PBC-DCB method that enables wafer center measurement. (**c**) PBC-DCB method that enables die-to-wafer measurement.

Blade insertion (BI) is a popular method for bonding strength measurement. It has several limitations to support the above measurement needs in for HB. (1) The measurement is limited at wafer edge locations. As shown in Figure 1a, BI measures the crack propagation length a after a blade is plunged into the gap between wafer bevels to initiate a crack along the bonding interface. The blade cannot reach the wafer center region due to the excessive distance [12]. (2) Since blade insertion relies on the bevel, it is difficult to apply to die-to-wafer HB samples. HB has two process schemes: wafer-to-wafer (W2W) bonding and die-to-wafer (D2W) bonding. Compared to W2W HB, which entered the industry earlier, D2W HB has emerged as an important format of HB for computing-memory die integration [9]. As shown in Figure 1b, there is no wafer bevel at the edge of the D2W samples. Therefore, a crack along the bonding interface cannot be initiated [13]. (3) The improvement in the measurement error is often limited by the rough front shape of the crack. The measurement of the crack propagation length a is based on infrared microscope imaging. The irregular shape can make the value of a ambiguous. Moreover, during the W2W bonding process, small edge voids are formed in the edge region due to the Joule–Thomson effect [14]. Thus, the results from the blade insertion method only reflect the bonding performance of the void-containing edge region and fail to capture the bonding strength uniformity across the entire wafer.

In this paper, we propose a novel method to overcome the above limitations. The method is referred to as the post-bonding crack-induced di-cantilever bending method (PBC-DCB). In the BI method, the crack along the bonding interface is initiated by the natural shape of the wafer bevel. Inspired by this, the PBC-DCB first creates a fabricated crack on the edge of the samples precisely aligned to the bonding interface, as shown in Figure 1b. Upon external force loading in the direction perpendicular to the bonding interface, the crack along the bonding interface can be initiated and propagated. By recording the loading displacement, the interface bonding energy can be calculated based on the di-cantilever beam model by Dai [15]. Test coupons can be cut from the center of the wafers from either W2W- or D2W-bonded samples, therefore enabling bonding strength measurement at any location of HB wafers in both formats.

## 2. Experimental Method and Theoretical Model

### 2.1. Experimental Methods and Sample Preparation

**Figure 2:**
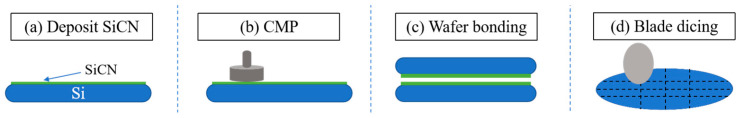
Schematic illustration of PBC-DCB sample preparation: (**a**) deposition of SiCN thin film via PECVD; (**b**) surface planarization of the wafer by CMP; (**c**) wafer bonding; (**d**) dicing the bonded wafers.

To overcome the shortcomings of existing bonding strength testing approaches and enable the reliable comprehensive assessment of the hybrid bonding integrity for both wafer-to-wafer and die-to-wafer configurations across the entire wafer area, the sample preparation process for the PBC-DCB test was designed as follows, as shown in Figure 2: Firstly, a layer of silicon carbonitride (SiCN) thin film was deposited on the 12-inch clean bare silicon substrate via the plasma-enhanced chemical vapor deposition (PECVD) technique, which serves as the bonding layer. The deposition parameters of PECVD are as shown in Table 1. Subsequently, the chemical mechanical polishing (CMP) process was employed to perform planarization treatment on the wafer surface, ensuring the surface roughness meets the bonding process requirements. CMP was performed using a 12-inch fully automated polisher. The final arithmetic surface roughness *R*_a_ is less than 0.3 nm, as measured by atom force microscope under standard tapping mode. After the completion of surface planarization, wafer bonding was conducted. Both the plasma activation and wafer bonding process were completed in an automatic fusion bonding system. Subsequently, the bonded wafer was diced by a wafer dicing saw, providing specimens with the dimensions required for the test.

**Figure 3:**
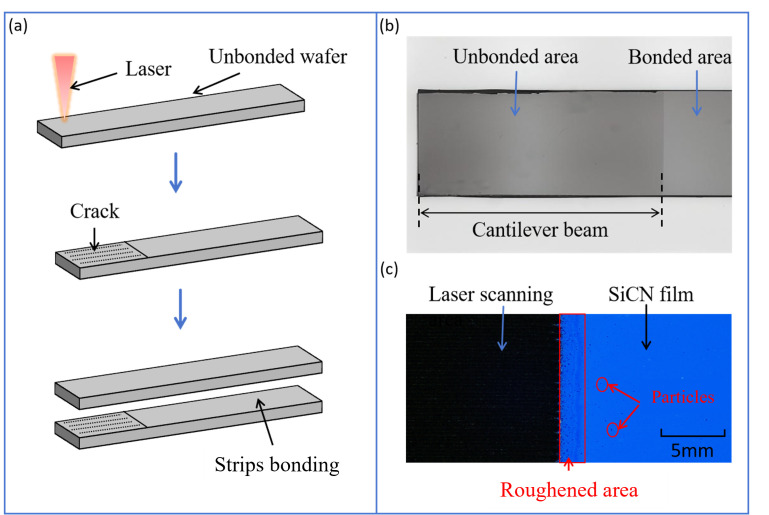
Schematic of pre-bonding crack initiation methods. (**a**) Introduction of a crack prior to bonding; (**b**) morphology image of the bonded specimen under infrared lens; (**c**) microscopic morphology image of the specimen after laser scanning.

The challenge in sample preparation lies in how to prepare a cantilever beam for testing. The traditional method involves creating a crack before bonding, which we refer to as the pre-bonding crack-induced DCB method. Figure 3a illustrates this process. Initially, the unbonded wafer is cut into testable strips of 40 × 5 mm^2^. A laser scans a portion of the surface and creates a roughened area. This area fails to form an effective bond during the subsequent bonding process due to its high surface roughness, as depicted in Figure 3b. A cantilever beam is thus formed above the area. This method inevitably introduces particle contamination during the laser scanning, therefore reducing the bonding strength, as compared to the real hybrid bonding processes where the surface is typically free of particles. As shown in Figure 3c, the edge region of the laser-scanned area exhibits high roughness.

To address the limitations of the traditional pre-bonding crack method, such as compromised bonding quality, poor uniformity, and unreliable measurements, the PBC-DCB introduces a novel post-bonding crack strategy, which significantly enhances the testing accuracy and repeatability. A key innovation here is that cracks are created after bonding via precision laser cutting aligned to the bonding interface, eliminating pre-bonding processing-induced contamination or surface damage. To enable accurate laser alignment, a “blade dicing—gradient grinding—chemical polishing” workflow is adopted: bonded wafers are first diced into 5 × 40 mm^2^ strips, then sequentially ground with fine-grit sandpapers, and finally polished with 1 μm and 50 nm slurries to achieve a smooth bonding layer surface. This treatment allows clear visualization of the bonding interface under a microscope, laying the foundation for precise laser crack positioning.

**Figure 4:**
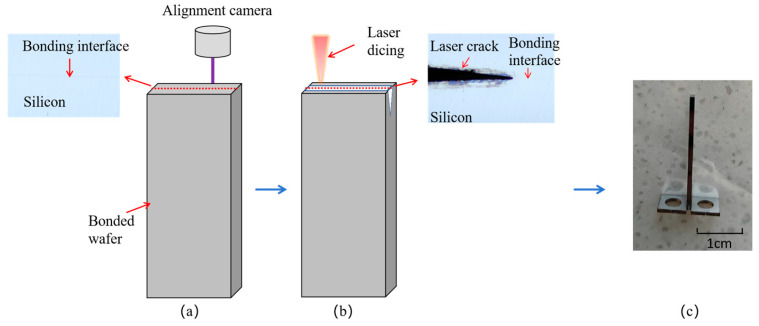
Schematic of post-bonding crack initiation methods. (**a**) Laser alignment and cutting process; (**b**) cutting crack coincides with the bonding interface; (**c**) actual image of the PCB-DCB specimen.

Figure 4a illustrates the laser alignment and cutting process, implemented using customized equipment. First, the bonding interface is identified via an alignment camera. The laser parameters are then adjusted to perform cutting with a crack depth of 1.5 mm. As shown in Figure 4b, the crack tip precisely coincides with the bonding interface. The portion of the coupon around the crack forms a di-cantilever beam, which is ready for testing after attaching pull rings (Figure 4c). The cutting laser has a frequency of 20 kHz, a pulse width of 40 μs, a processing count of 100 times, and a moving speed of 10 mm/s.

### 2.2. PBC-DCB Model

**Figure 5:**
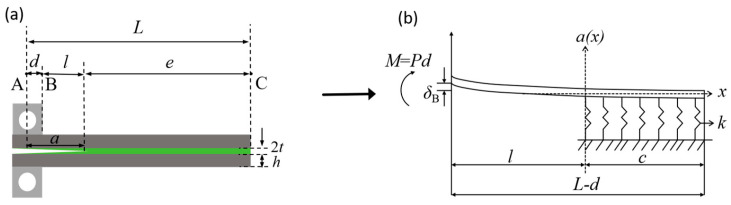
(**a**) Schematic diagram of the PBC-DCB model. (**b**) Schematic diagram of the elastic foundation beam model.

To quantitatively evaluate the bonding strength and establish a clear correlation between the load–displacement behavior and the energy release rate, the PBC-DCB model is derived based on the double cantilever beam (DCB) fracture mechanics theory with targeted adaptations, and its geometric configuration and testing principles are described as follows. The geometric shape of the test specimen is shown in Figure 5. In this figure, *a* denotes the crack length, *d* denotes half the length of the metal pull ring, *l* denotes the distance from the end of the metal pull ring to the front of the initial crack, *e* denotes the distance from the front of the initial crack to the end of the sample, and *L* denotes the distance from the load point to the end of the sample.

During testing, stainless-steel nuts are glued to both sides of the test coupons as the pull rings. The pull rings are then connected to the hooks of a tensile tester which can record load–displacement (*l-d*) curves accurately. The crack grows along the bonding layer from the fabricated crack. The specimen is loaded under a constant displacement rate mode until local crack propagation is observed, which causes the load in the *l*-*d* curves to drop.

According to the content of fracture mechanics, it is known that the energy release rate per unit area for interfacial crack propagation in the PBC-DCB model is a function of the derivative of compliance *C* with respect to crack length *a*.

(1) $$G=\frac{P^{2}}{2b}\cdot \frac{dC}{da}$$

The calculation of the bonding strength is derived from the PBC-DCB analytical model, according to Dai [15], and the Euler–Bernoulli equation and Winkler theory [16]; the model studied in this project is actually a Si-SiCN-Si sandwich structure. Therefore, the Si wafers can be regarded as the beams, and the surface SiCN layers can be regarded as the intermediate adhesive layers; the expressions for the compliance and energy release rate are as follows:

(2) $$C=\frac{1}{E_{s}I_{2}\lambda_{0}^{3}}\left(\frac{\lambda_{0}^{3}\left(2a^{3}-a^{2}d-2ad^{2}+d^{3}\right)}{3}+\lambda_{0}\left(2a-d\right)+2\lambda_{0}^{2}a\left(a-d\right)+1\right)+\frac{3(a-d)}{bG_{s}h}$$

(3) $$G=\frac{P^{2}}{2E_{s}I_{2}b\lambda_{0}^{3}}\left(\frac{\lambda_{0}^{3}(6a^{2}-2ad-2d^{2})}{3}+2\lambda_{0}+2\lambda_{0}^{2}\left(2a-d\right)\right)+\frac{3P^{2}}{2b^{2}G_{s}h}$$

*λ*_0_ is a characteristic parameter of the test model, which is used to quantify the matching relationship between the bending stiffness of the beam and the foundation support stiffness. Based on the above derivation, the compliance “*C*” and the bonding strength “*G*” can be calculated using the peak load “*P*” and displacement “*δ*” obtained from the tensile cyclic testing. The physical values used in the calculation are shown in Table 2.

### 2.3. Crack Propagation Process of PBC-DCB

**Figure 6:**
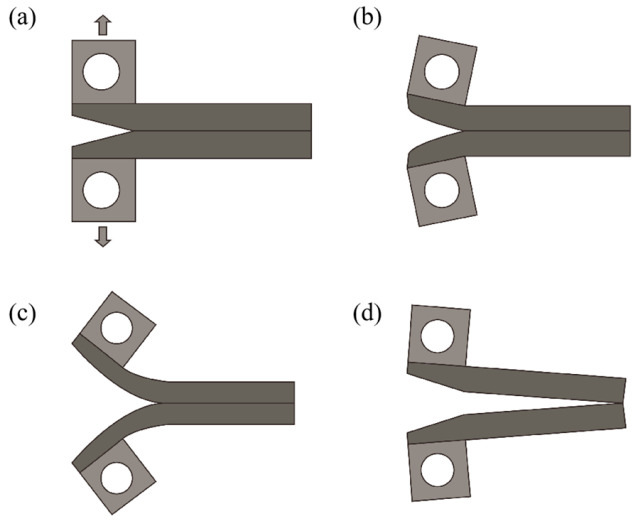
Crack propagation process: (**a**) load is applied to pull rings on upper and lower beams; (**b**) upper and lower beams start to bend; (**c**) crack propagates from the initial location; (**d**) upper and lower beams completely separate.

In the tensile testing process, the testing speed of the tensile force was set at 0.1 mm/min. The sensor accuracy of the tensile tester is 0.02% of full scale, which ensures the accuracy of the measurement data. This is because, at a low testing speed, crack propagation can be assumed to be in a quasi-static state. The increases in the deflection of the cantilever beam can achieve a relative equilibrium with the crack propagation. The experiments were performed in a glovebox with pure N2 atmosphere (moisture level < 0.1 ppm) to prevent moisture corrosion of the interface during the tests [17]. The crack propagation process is shown in Figure 6. Upon vertical loading, the fracture mode belongs to pure Mode I opening crack propagation, and the *l-d* curve exhibits linear elastic characteristics. As shown in Figure 6a,b, when the load-induced “*G*” exceeds the interfacial fracture energy (“*G_c_*”), the crack propagates from the post-bonding crack tip towards the bonding interface, as shown in Figure 6c. The load–displacement curve decreases. At this point, the main difference from the blade insertion method is that the crack propagation length is calculated from the peak load and compliance, eliminating the measurement error of the crack propagation from the microscope image in the blade insertion method. Subsequently, the constraint on the cantilever beam is released, the load decreases, and the process is repeated for several cycles. Cyclic load–unload is performed with the same step size until the whole interface is separated.

## 3. Experimental Results and Discussion

### 3.1. Load–Displacement Behavior of PBC-DCB

**Figure 7:**
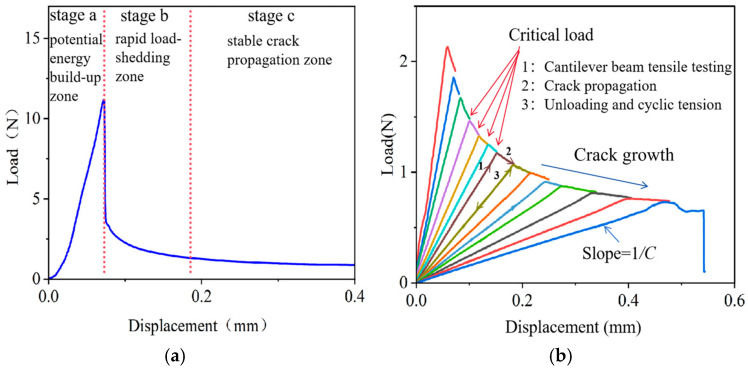
Load–displacement curve of a tensile cantilever beam. (**a**) Load–displacement curve of single tensile test; (**b**) load–displacement curve of cyclic tensile test. Various colors are used for differentiating the curves in each cycle.

The load–displacement curve of a single tensile cantilever beam stretching can be divided into three stages, as shown in Figure 7a. Stage a: The cantilever beam bends, and the load begins to increase linearly. At this point, the work performed by the external force is converted into the elastic strain energy of the specimen. Stage b: As the tensile force increases and reaches the threshold for bonding layer cracking, the strain energy stored in the interface instantaneously exceeds the fracture energy “*G_c_”*, causing the crack to propagate. Since the crack propagation rate is much faster than the displacement loading rate at this time, the load drops rapidly. This stage is referred to as the rapid load drop zone. Stage c: The crack propagation rate and the displacement rate of the cantilever beam reach a relative equilibrium. The compliance of the cantilever beam increases nonlinearly with the crack length. Under constant-rate loading of the external force, the energy release rate and the interfacial fracture energy maintain a continuous dynamic balance. The crack propagation rate matches the loading rate, and the driving load decreases steadily as the crack extends stably. This stage is referred to as the plateau zone.

To enhance the accuracy and measurability of bonding strength characterization, the cyclic DCB testing method is adopted, as shown in Figure 7b. By employing a “loading → resetting → repeating loading” cyclic pattern, the displacement beam is controlled to return to its initial state after the load reaches its peak, at which point both the load and displacement are reset to zero. The process is repeated to obtain multiple load–displacement curves, each representing the bonding strength at different locations within the sample. Since the crack growth in the rapid load drop zone is unstable and propagates rapidly, the corresponding bonding strength values are unstable and show a rapid downward trend. Therefore, only the bonding strengths corresponding to all the load–displacement curves in the plateau zone are calculated, and the average value is taken to evaluate the bonding strength of the sample.

### 3.2. Load–Displacement Curve Differences of Pre- vs. Post-Bonding Crack Methods

**Figure 8:**
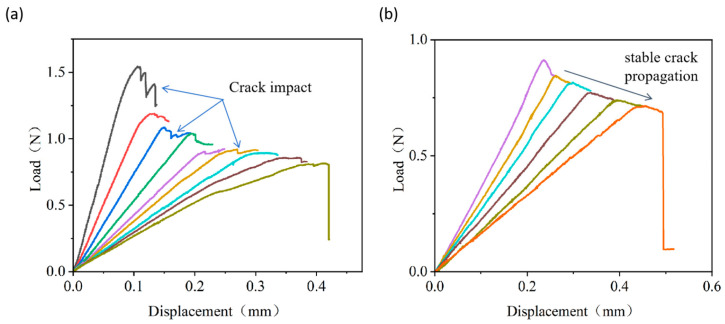
(**a**) Pre-bonding introduced crack cyclic tension di-cantilever beam curve of SiCN; (**b**) post-bonding introduced crack cyclic tension di-cantilever beam curve of SiCN. Various colors are used for differentiating the curves in each cycle.

The results of PBC-DCB are compared to those from conventional DCB to further demonstrate its advantage. The conventional DCB has to introduce cracks before bonding, which inevitably brings debris contamination from laser cutting to the bonding surface and induces subsequent bonding voids. As shown in Figure 8a, during the descending segment of crack propagation, the load–displacement curve exhibits significant fluctuation. The method of introducing cracks after bonding does not affect the bonding surface and thus does not impact the bonding quality. The load–displacement curve shows a smooth descending phase, as illustrated in Figure 8b.

### 3.3. Impact of Pull Ring/Sample Length Ratios

**Figure 9:**
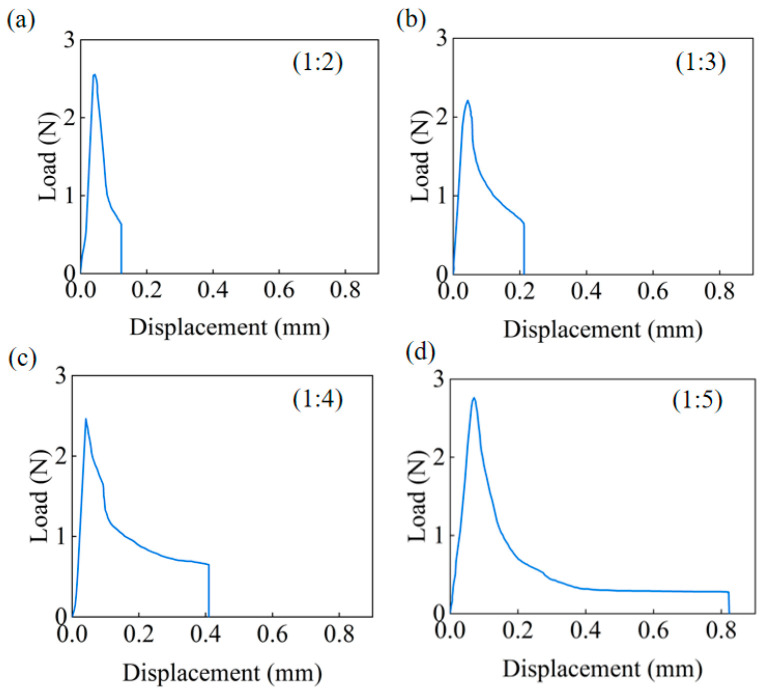
Load–displacement curves for different pull ring-to-specimen length ratios. (**a**) 1:2 (**b**) 1:3. (**c**) 1:4. (**d**) 1:5.

To optimize the PBC-DCB test configuration for accurate bonding strength evaluation, the effect of the pull ring/sample length ratios on the test outcomes is examined in this section. With the pull ring length fixed, the study investigates the influence of the pull ring/sample length ratios of 1:2, 1:3, 1:4, and 1:5 on the PBC-DCB test results. Single DCB tests are performed on samples with different pull ring/sample length ratios, and the load–displacement curves are compared, as shown in Figure 9.

When the ratio of the length of the pull ring to the specimen is relatively large (such as 1:2, 1:3), a decrease in the effective length fraction of the cantilever beam will significantly increase the bending stiffness according to the formula for the bending stiffness of a cantilever beam. During the loading process, the elastic strain energy accumulates rapidly. When the energy release rate for crack propagation reaches the interfacial fracture energy, the stored high strain energy is instantaneously released, which is manifested as a “sharp peak and sudden drop” in the load–displacement curve. Under this condition, the proportion of the stable load decrease segment (corresponding to the steady-state crack propagation stage) in the total displacement is less than 30%, and the measurable data effectively used for bond strength calculation account for an extremely low proportion.

As the length ratio increases (such as 1:4, 1:5), the effective length L of the cantilever beam increases, and the bending stiffness decreases. When the crack initiates, the rate of work by the external force and the rate of energy dissipation due to crack propagation are more likely to maintain a dynamic equilibrium. This results in the stable load decrease segment accounting for more than 60% of the total displacement, making the crack propagation behavior closer to a steady state and providing a good basis for the measurability of the PBC-DCB.

### 3.4. Fracture Interface Characterization

**Figure 10:**
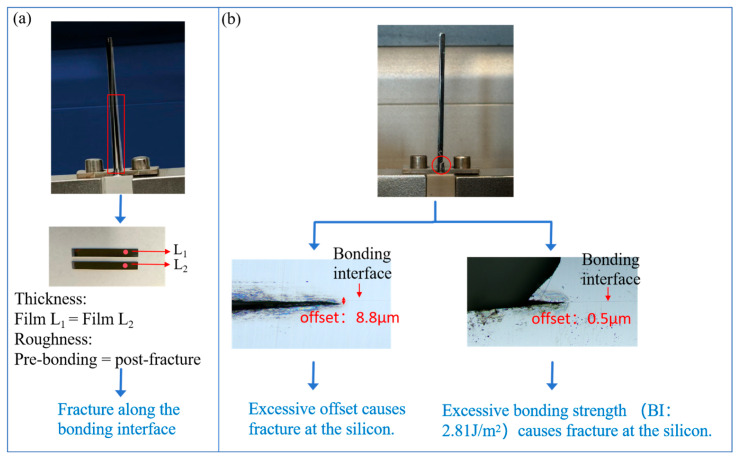
Two fracture modes of fusion bonding. (**a**) Fracture along the bonding interface; (**b**) excessive offset causes fracture at the silicon, and excessive bonding strength causes fracture at the silicon.

To clarify the fracture mechanism and ensure test reliability, this section characterizes the fracture interface by investigating the effects of pre-crack cutting deviation and bonding strength on the fracture location. During the laser pre-crack cutting process, there may be deviations between the pre-crack and the bonding interface due to cutting precision issues. The magnitude of these deviations can severely affect the success rate of the tensile testing. Based on thirty sets of experimental data, when the bonding strength of the sample is lower than the bulk fracture energy of silicon (2.5 J/m^2^), and the cutting offset is within 5 μm from the bonding interface, the success rate would be about 70%. When the bonding strength of the sample is higher than the bulk fracture energy of silicon, bulk silicon fracture is likely to occur. In this case, the cutting offset needs to be controlled within 2 μm to maintain the same success rate. To validate these empirical rules, two wafers were bonded and tested. Each wafer has a layer of SiO_2_ thin film on bare Si substrates. The thickness of the SiCN thin film on both wafers is 214 nm. After bonding, PBC-DCB testing was performed with the post-bonding crack offset controlled within 5 μm from the bonding interface. As shown in Figure 10a, the sample was successfully pulled apart. The film thickness measurement demonstrated that the thickness of the film on the top die (*L*_1_) and bottom die (*L*_2_) were 214.41 nm and 214.13 nm, respectively. The pre-bonding roughness is basically consistent with the post-fracture roughness. The thickness and roughness of the films on both sides of the fracture is basically the same, which indicates that the fracture occurred at the bonding interface. In Figure 10b, the sample fractured within the bulk silicon. The reason is that the cutting offset was too large or the bonding strength of the sample itself was higher than the fracture energy of bulk silicon.

**Figure 11:**
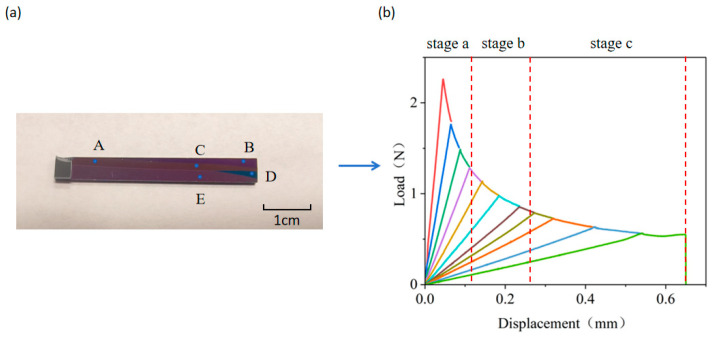
(**a**) SiCN-SiCN bonded fracture interface. (**b**) Corresponding displacement–load curve.

In another demonstration, Si wafers with SiCN thin film on top were bonded. The deposition of the SiCN film was deliberately adjusted so that there were defects within the film. After PBC-DCB testing, it was found that there were multiple fracture interfaces (Figure 11). After testing the film thickness with a film thickness meter, it was found that the thickness of the films at different locations A–E in Figure 11 were not the same. This is because during the tensile testing, the crack always propagates along the weakest interface, as this requires the least amount of energy. For this batch of bonded samples, the internal adhesion strength of the thin film was less than the bonding strength of the bonding interface, which could lead to an underestimation of the measured bonding strength.

### 3.5. Comparison of the Bonding Strength Measurement Results Between BI and PBC-DCB

**Figure 12:**
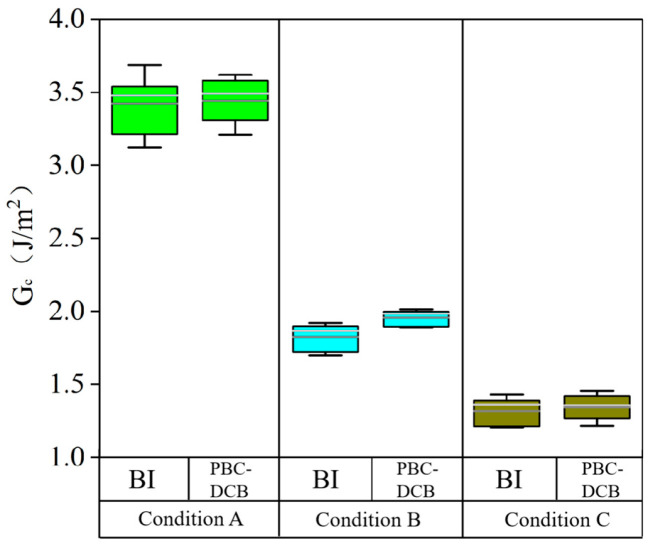
Bonding strength results comparison between PBC-DCB and BI.

The PBC-DCB method was validated by comparing its results with the BI results. Si wafers with SiO_2_ thin films were bonded. The crystal orientation of the Si substrates (<100>) and location in the wafer were kept identical during comparison. To further demonstrate the resolution of both methods, the wafers went through three different activation and annealing temperature conditions. To ensure consistent crystal orientation, the same position and direction were selected for both methods during testing. The measurement results are shown in the box plot in Figure 12. The results indicate that the numerical deviation between the two methods is within 5%, and the measured bonding strength values under different conditions exhibit the same trend, confirming the reliability of the PBC-DCB method. After *t*-test analysis, the *p*-values of all three groups were higher than 0.05, demonstrating that there was no significant difference in the means under the three conditions. It can be seen from the box plot that the standard deviation of the PBC-DCB method is always smaller than those of BI, confirming a smaller test error of the PBC-DCB method. In addition, the test results of the PBC-DCB method are slightly higher than those of the blade insertion method.

### 3.6. Comparison of the Bonding Strength Between SiCN and SiO_2_ Under the Same Conditions

**Figure 13:**
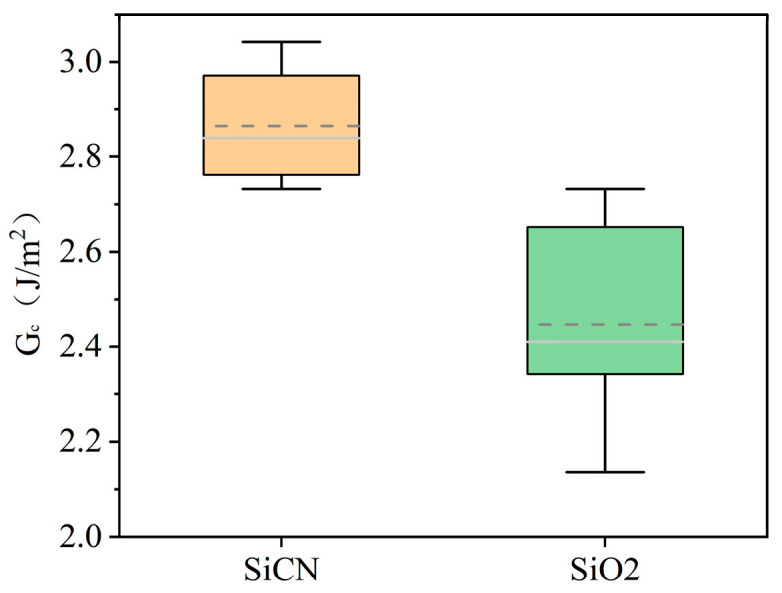
Comparison of bonding strength between SiCN and SiO_2_ under different activated gas conditions.

The resolution of PBC-DCB is further demonstrated by using different bonding film materials. In addition to SiO_2_, SiCN is another major type of dielectric film for HB. SiCN has a higher mechanical strength and is more resistant to the physical impact of chemical mechanical polishing (CMP), reducing the process defects [18]. It also has a stronger ability to inhibit Cu diffusion. It is suitable for high-density interconnection with pitch below 1 μm. In the literature, a higher bonding strength by SiCN low-temperature annealing has been reported [19,20,21,22]. The bonding strengths of SiCN and SiO_2_ were tested under the same activation gas and annealing temperature conditions. The test results are shown in Figure 13. Among the multiple sets of data tested, the average bonding strength of SiCN was 2.87 J/m^2^, while that of SiO_2_ was 2.45 J/m^2^. The bonding strength of SiCN was 17.34% higher than that of SiO_2_, consistent with trends in the literature using BI methods. Under the optimized parameters of this experiment, the intrinsic stresses of SiCN and SiO_2_ deposited by Plasma Enhanced Chemical Vapor Deposition (PECVD) are relatively low and all within the controllable range. After planarization, the film uniformity is within 5%; thus, the influence of the film’s intrinsic stress and thickness mismatch on the bonding strength can be excluded. It is worth noting that the standard variation of the SiCN bonding strength is narrower, reflecting that the SiCN dielectric layer has better uniformity in large-size wafer bonding. This is crucial for improving the yield of mass production-level 3D integration processes.

### 3.7. Measurement Results of Bonding Strength in Different Regions of Wafer Bonding

**Figure 14:**
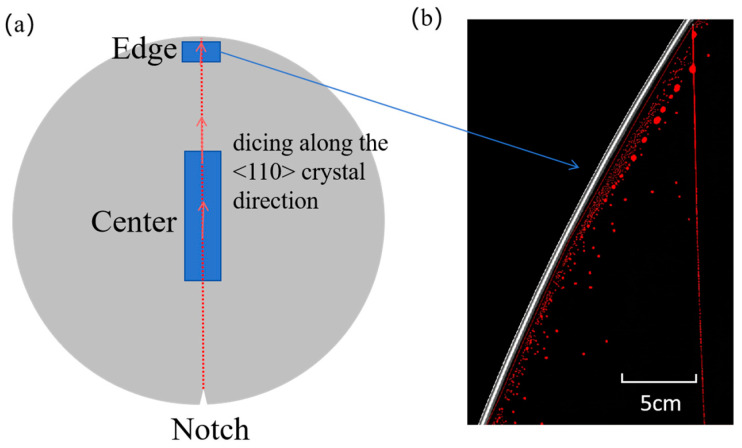
Bonding strength testing regions: (**a**) wafer cutting position schematic; (**b**) edge region C-SAM scan image.

**Figure 15:**
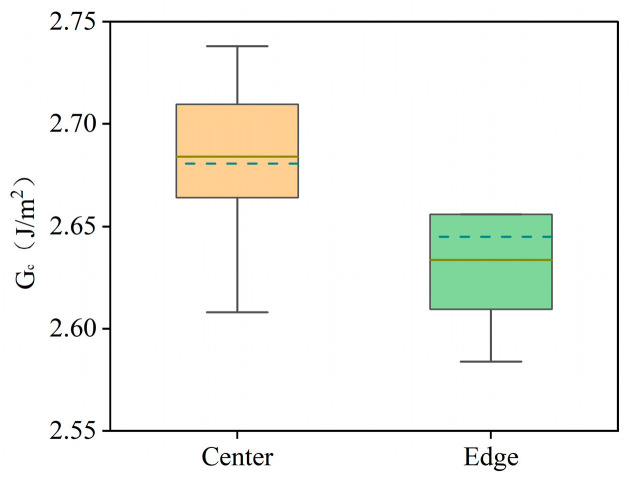
Comparison chart of bond strength in center and edge regions.

To demonstrate the advantage of PBC-DCB, which is that it can measure both the center and edge region of W2W bonding samples, the center and edge regions of the same bonded wafer pair were measured. The measurement locations and cutting directions are shown in Figure 14a, ensuring that the cutting and testing directions are aligned with the <110> crystal orientation of Si substrates. The measurement results of the bonding strength are shown in Figure 15, with the average bonding strength in the center region being 2.68 J/m^2^ and that in the edge region being 2.64 J/m^2^. The bonding strength in the center region is 1.52% higher than that in the edge region. Figure 14b presents a representative C-SAM scan image of the wafer edge region. The lower bonding strength in the wafer edge region is likely attributed to stress concentration during bonding and the formation of small voids induced by the Joule–Thomson effect [13].

## 4. Conclusions

This study proposes a novel post-bonding crack-induced di-cantilever bending (PBC-DCB) method to address the limitations of the traditional blade insertion (BI) technique in evaluating the hybrid bonding strength. By systematically optimizing the sample preparation procedures and testing parameters and deriving the load–displacement-bonding strength relationship based on fracture mechanics, the PBC-DCB method achieves reliable quantitative characterization of bonding strength for both wafer-to-wafer (W2W) and die-to-wafer (D2W) hybrid bonding configurations.

(1) The core contribution of PBC-DCB lies in its ability to overcome the inherent drawbacks of BI. Specifically, it enables bonding strength measurement at any location on the wafer—including the center region—and is fully applicable to D2W samples that lack bevel structures. Notably, the method’s innovative post-bonding laser crack strategy eliminates contamination and surface damage caused by pre-bonding processing, thereby ensuring the integrity of the bonding interface. This design fundamentally resolves the critical issue, where BI fails to reflect the overall bonding uniformity of wafers.
(2) Experimental validation further confirms the reliability of PBC-DCB: its measurements are highly consistent with those of BI, showing minimal deviation and significantly better repeatability. Additionally, parameter optimization enhances the test accuracy—with an optimal pull ring-to-sample length ratio, the load–displacement curve exhibits a stable descending segment, providing sufficient valid data for precise bonding strength calculation. Furthermore, fracture interface characterization validates that PBC-DCB accurately captures interfacial fracture behavior, avoiding misjudgments caused by improper crack positioning.
(3) Regarding application potential: PBC-DCB can effectively distinguish differences in bonding performance between various dielectric materials, as well as between the center and edge regions of a wafer. Such a capability provides critical technical support for material selection, process optimization, and yield improvement in hybrid bonding—especially in high-density heterogeneous integration scenarios, such as AI chips. Overall, as a reliable testing tool, PBC-DCB holds broad prospects for application in both industrial production quality control and scientific research on hybrid bonding technology.

Characterization of the interface involving Cu pads is ongoing. Due to the ductility of copper, the crack may deviate from the dielectric interface with the presence of copper. However, we believe the general idea that a post-bonding crack helps initiate an interface crack should still apply to the case with a Cu pad.

## Figures and Tables

**Table 1:** Specific parameters for PECVD deposition of SiCN.

HF/W	Spacing	Pressure/Torr	NH_3_ Flow Rate/Sccm	N2 Flow Rate/Sccm	TMS Flow Rate/Sccm
650	290	3.7	725	1200	350

**Table 2:** Definitions and parameter of physical quantities in the PBC-DCB test model.

Parameter	Symbol (Unit)	Value
Young’s modulus of Si	*E*_s_ (*GPa*)	169 (<110>)
		128 (<100>)
Poisson’s ratio of Si	*v*_s_	0.064 (<110>)
		0.28 (<100>)
Young’s modulus of SiCN	*E*_0_ (*GPa*)	200
Poisson’s ratio of SiCN	*V*_0_	0.21
The thickness of Si-1	*h* (μm)	775
The thickness of Si-2	*h* (μm)	775
The thickness of SiCN/SiO_2_-1	*t* (nm)	200/300
The thickness of SiCN/SiO_2_-2	*t* (nm)	200/300
The width of the wafer	*b* (mm)	4~5
Half-length of nut	*d* (mm)	0.60~0.65

## Data Availability

The original contributions presented in this study are included in the article/Supplementary Material. Further inquiries can be directed to the corresponding author.

## Supplementary Materials

The following supporting information can be downloaded at: https://www.mdpi.com/article/10.3390/mi16121304/s1.
