# Supplementary material for: Post-Bonding Crack-Induced Di-Cantilever Bending (PBC-DCB): A Novel Method for Quantitative Evaluation of Bonding Strength for Wafer-to-Wafer and Die-to-Wafer Hybrid Bonding
Source: Micromachines (Basel). 2025 Nov 21;16(12):1304. doi: 10.3390/mi16121304 (PMC12734516; doi:10.3390/mi16121304)
Supplement: Supplementary file 1 [file micromachines-16-01304-s001.zip › micromachines-3974382-supplementary.pdf]

Supporting information

Post-Bonding Crack-Induced Di-Cantilever Bending (PBC-DCB): A Novel Method for Quantitative Evaluation of Bonding Strength for Wafer-to-Wafer and Die-to-Wafer Hybrid Bonding

Tianze Zheng [1,#], Yuan Xu [1,#], Cong Mei [1], Yingjie Chen [1], Liu Chang [1], Gangli Yang [1], Qiuhan Hu [2],Tailong Shi[1], Yuan Yuan[3] ,Zongguang Yu[3] and Liyi Li [1,*]

1 School of Integrated Circuits, Southeast University, Wuxi 214000, People's Republic of China; 220236463@seu.edu.cn (T. Z.); 220226140@seu.edu.cn (Y. X.);230229587@seu.edu.cn(M.C.) siriusirs@163.com(C. Y.) 230239483@seu.edu.cn (C. L.); 230238438@seu.edu.cn(G.Y.);101013381@seu.edu.cn (T.S.)

2 School of Advanced Technology, Xi'an Jiaotong-Liverpool University, Suzhou 215123, People's Republic of China, Qiuhan.Hu23@student.xjtlu.edu.cn.

3 The 58th Research Institute of China Electronics Technology Group Corporation, Wuxi 214000, People's Republic of China, 15629070655@163.com (Y.Y.) ; yuzg58@163.com (Z.Y.)

# These authors contributed to the work equally and should be regarded as co-first authors.

* Correspondence: liyi_li@seu.edu.cn

Based on Dai's model, the following is the derivation of the calculation formula for the PBC-DCB model. For details, refer to the paper https://doi.org/10.1109/6144.833049.

S1. PBC-DCB Model

To quantitatively evaluate the bonding strength and establish a clear correlation between load-displacement behavior and energy release rate, the PBC-DCB model is derived based on the double cantilever beam (DCB) fracture mechanics theory with targeted adaptations, and its geometric configuration and testing principles are described as follows. The geometric shape of the test specimen is shown in Figure 5. In this figure, a denotes the crack length; d denotes half the length of the metal pull ring; l denotes the distance from the end of the metal pull ring to the front of the initial crack; e denotes the distance from the front of the initial crack to the end of the sample; and L denotes the distance from the load point to the end of the sample.

During testing, stainless steel nuts are glued at both sides of the test coupons as the pull rings. The pull rings are then connected to hooks of a tensile tester which can record load-displacement (l-d) curves accurately. The crack grows along the bonding layer from the fabricated crack. The specimen is loaded under a constant displacement rate mode until local crack propagation is observed, which causes the load in the l-d curves to drop. The calculation of bonding strength is derived from DCB analytical model according to Dai . The model is briefly described below.

The model is divided into two parts according to the force trend. The first part is from the load point to the edge of the pull ring (segment AB), and the second part is from the edge

of the pull ring to the edge of the cantilever beam (segment BC). The deflection at the load point (point A) is equal to the sum of the deflections of these two parts. The deflection of the first part accounts for less than 10% and can be neglected. Therefore, the total deflection at point A is:

$$\delta_A = \delta_B^{II} + d\phi_B^{II} \tag{S1}$$

Where, "δ_A" is the deflection at the load point; "δ_B^{II}" is the deflection of the second part; "φ_B^{II}" is the rotation of the second part at point B.

According to the Euler-Bernoulli equation and Winkler theory, each cantilever on both sides of segment BC in the PBC-DCB model is treated as a beam structure, with part of the region free and the rest supported by an elastic foundation, as shown in Figure 5. Then, the governing differential equation for the beam deflection α(x) can be established by the following equation :

$$\frac{d^4\alpha(x)}{dx^4} + 4\lambda^4 H(x)\alpha(x) = 0 \tag{S2}$$

$$H(x) = \begin{cases} 1 & x>0 \\ 0 & x<0 \end{cases} \tag{S3}$$

$$\lambda^4 = \frac{k}{4E_x I} \tag{S4}$$

Where I is the section moment of inertia of the beam at the corresponding position, and λ is a function of the foundation modulus, elastic modulus, and section moment of inertia. H is a stepwise function separating the movable beam AB and the fixed foundation BC. E_x refers to the elastic modulus of the beam in the x-axis direction. The corresponding boundary conditions are:

$$M_l = E_x I_x \alpha''(-l) = P\cdot d \tag{S5}$$

$$M_r = E_x I_x \alpha''(c) = 0 \tag{S6}$$

$$S_l = E_x I_x \alpha'''(-l) = P \tag{S7}$$

$$S_r = E_x I_x \alpha'''(c) = 0 \tag{S8}$$

M_l and M_r denote the bending moments at the left end and right end of the beam, respectively; S_l and S_r denote the shear forces at the left end and right end of the beam, respectively; and P is the load acting on the beam.

By combining the beam deflection equation with the boundary conditions and considering the correction for shear force according to the elastic theory of Timoshenko and Goodier, the expression for the deflection at point A can be obtained:

$$\delta_A = \delta_B^{II} + d\varphi_B^{II} = \frac{P}{2E_x I\lambda^3}\left(\frac{\lambda^3(2l^3+3l^2d)}{3} + A\lambda a + B\right) + \frac{3lP}{2bG_{xy}h} \tag{S9}$$

In the formula, b is the width of the cantilever beam in the model; h is the thickness of the cantilever beam on one side in the DCB model; and G_{xy} is the shear modulus of the cantilever beam in the model. The unknown in the above equation is the stiffness k. The model in this study is a symmetric structure with the top and bottom both being cantilever beams composed of the bonding layer and silicon wafers, so the foundation modulus of the top and bottom are the same. The expressions for the foundation modulus of the top and bottom dies are:

$$k_{top} = k_{bottom} = \frac{1}{\frac{1}{k_1} + \frac{1}{k_2}} \tag{S10}$$

Where, "k_{top}" is the stiffness of the top dies; "k_{bottom}" is the stiffness of the bottom; "k_1" is the silicon substrates; "k_2" is the stiffness of the bonding layer; In the PBC-DCB model, the role of the elastic foundation is to compensate for the missing half of the beam. Thus, the

foundation modulus here is not independent. Instead, its value is related to the half-height and width of the beam, and is linked through the average transverse strain and transverse stress in the beam. The specific relationship is as follows:

$$\sigma_y^{(1)}=\frac{k_1\alpha(x)}{b} \qquad (S11)$$

$$\varepsilon_y^{(1)}=\frac{\alpha(x)}{h/2} \qquad (S12)$$

$$\sigma_y^{(1)}=E_y^{(1)}\varepsilon_y^{(1)} \qquad (S13)$$

In the above equations, $\sigma_y^{(1)}$ and $\varepsilon_y^{(1)}$ are the transverse stress and transverse strain of the beam, respectively. $E_y^{(1)}$ is the elastic modulus of the beam in the y-direction.

According to Equations S2-S11, S2-S12 and S2-S13, the expression of k_1 can be obtained:

$$k_I=\frac{2E_y^{(1)}b}{h} \qquad (S14)$$

By considering the stress-strain relationship of the adhesive layer, the expression of k_2 can be obtained. Assuming that the axial strain in the adhesive layer plane is suppressed $\varepsilon_y^{(2)}$, the following relationship can be obtained according to Hooke's law under plane stress conditions and the foundation model established above:

$$\sigma_y^{(2)}=\frac{2E_y^{(2)}b}{1-v_{xy}^{(2)}v_{yx}^{(2)}}\varepsilon_y^{(2)} \qquad (S15)$$

$$\varepsilon_y^{(2)}=\frac{\alpha(x)}{t} \qquad (S16)$$

$$\sigma_y^{(2)}=\frac{k_2\alpha(x)}{w} \qquad (S17)$$

In the above equations, $\sigma_y^{(2)}$ and $\varepsilon_y^{(2)}$ are the transverse stress and transverse strain of the adhesive layer, respectively; $E_y^{(2)}$ is the elastic modulus of the adhesive layer in the y-direction; $v_{xy}^{(2)}$ and $v_{yx}^{(2)}$ are the Poisson's ratios of the adhesive layer; w is the width of the adhesive layer; and t is half the thickness of the adhesive layer.

According to Equations S2-S15, S2-S16 and S2-S17, the expression of k_2 can be obtained:

$$k_2=\frac{E_y^{(2)}}{1-v_{xy}^{(2)}v_{yx}^{(2)}}\cdot\frac{w}{t} \qquad (S18)$$

Based on this, the deflection at load point A can be determined.

$$\delta=\delta_A^t+\delta_A^b=\frac{P}{E_xI_i\lambda_i^3}\left(\frac{\lambda_i^3(2a^3-a^2d-2ad^2+d^3)}{3}+A_i\lambda_ia+B_i\right)+\frac{3(a-d)P}{bG_{xy}h} \qquad (S19)$$

Thus, the expression for the compliance "C" is derived.

$$C=\frac{\delta}{P}=\frac{1}{E_xI_i\lambda_i^3}\left(\frac{\lambda_i^3(2a^3-a^2d-2ad^2+d^3)}{3}+A_i\lambda_ia+B_i\right)+\frac{3(a-d)}{bG_{xy}h} \qquad (S20)$$

The DCB model studied in this project is actually a Si-SiCN-Si sandwich structure. Therefore, the Si wafers can be regarded as the beams, and the surface SiCN layers can be regarded as the intermediate adhesive layers. By treating Si and SiCN as homogeneous materials with equal widths, the relevant variables in the above expressions can be further simplified:

$$E_x=E_y=E_s \qquad (S21)$$

$$E_y^2=E_0^2 \qquad (S22)$$

$$G_{xy}=G_s \tag{S23}$$

$$G_s=\frac{E_s}{(1+v_s)} \tag{S24}$$

$$v_{xy}^2=v_{yx}^2=v_o \tag{S25}$$

$$v_{xy}^3=v_{yx}^3=v_s \tag{S26}$$

$$I_t=I_b=I_2 \tag{S27}$$

Among them, E_s and E_0 represent the Young's modulus of Si and silicon SiCN, respectively; Grepresents the shear modulus of silicon; v_0 represents the Poisson's ratio of SiCN; v_s represents the Poisson's ratio of silicon; and I_2 represents the moment of inertia of the cross-section of segment BC in the PBC - DCB model.

According to the content of fracture mechanics, it is known that the energy release rate per unit area for interfacial crack propagation in the PBC - DCB model is a function of the derivative of compliance with respect to crack length.

$$G=\frac{P^2}{2b}\cdot\frac{\mathrm{d}C}{\mathrm{d}a} \tag{S28}$$

For the SiCN-SiCN bonded wafer, the SiCN film deposited on the Silicon surface has a certain thickness and cannot be ignored in the calculation process. In this model, both the upper and lower beams can be regarded as a spring system in which Si beams are connected in series with SiCN adhesion layers. Then, the foundation modulus in this bonding model is given by the following formula:

$$k_1=\frac{2E_sb}{h} \tag{S29}$$

$$k_2=\frac{E_0}{1-v_0^2}\cdot\frac{b}{t} \tag{S30}$$

$$k_t=k_b=\frac{1}{1/k_1+1/k_2} \tag{S31}$$

Combined with Equations (S2-S4), the expression for is:

$$\lambda_0{}^4=\frac{3E_0}{(1-v_0^2)E_Sth^3} \tag{S32}$$

The expressions for compliance and energy release rate are as follows:

$$C=\frac{1}{E_sI_2\lambda_0^3}\left(\frac{\lambda_0^3\left(2a^3-a^2d-2ad^2+d^3\right)}{3}+\lambda_0(2a-d)+2\lambda_0^2a(a-d)+1\right)+\frac{3(a-d)}{bG_sh} \tag{S33}$$

$$G=\frac{P^2}{2E_sI_2b\lambda_0^3}\left(\frac{\lambda_0^3(6a^2-2ad-2d^2)}{3}+2\lambda_0+2\lambda_0^2(2a-d)\right)+\frac{3P^2}{2b^2G_sh} \tag{S34}$$

λ_0 is a characteristic parameter of the test model, which is used to quantify the matching relationship between the bending stiffness of the beam and the foundation support stiffness. Based on the above derivation, the compliance "C" and the bonding strength"G" can be calculated using the peak load "P" and displacement "δ" obtained from the tensile cyclic testing. The physical values used in the calculation are shown in Table 2.
